# Propofol and priapism

**DOI:** 10.4103/0253-7613.68430

**Published:** 2010-08

**Authors:** Subramanian Senthilkumaran, Sweni Shah, Ganapathysubramanian, Namasivayam Balamurgan, Ponniah Thirumalaikolundusubramanian

**Affiliations:** Sri Gokulam Hospitals and Research Institute, Salem, TamilNadu, India; 1 University of Debrecen, Medical and Health Science Center, Debrecen, Hungary; 2 Chennai Medical College and Research Center, Irungalur, Trichy, India

**Keywords:** Diisopropylphenol, early recognition, low-flow priapism, mechanisms, propofol, rechallenge

## Abstract

Propofol-induced priapism in a 25-year-old male confirmed by rechallenge is reported for its rarity and to create awareness among practitioners, because propofol is used frequently in India for the induction and maintenance of anesthesia or sedation. The probable mechanisms are highlighted. Because propofol causes low-flow priapism, early alleviation is essential to minimize and/or avert the long-term complications.

## Introduction

Propofol (2, 6 diisopropylphenol) is a commonly used intravenous anesthetic and sedative agent in the critical care unit because ofits favorable properties like quick onset of action, early recovery with thediscontinuation of drug and absence of significant nausea.[1,2] The most commonly reported adverse events with this drug are the development of respiratorydepression, hypotension, pain, infection during infusion and myoclonus. Propofol has been reported to cause sexual hallucinations during and after sedation, but priapism was documented rarely in some individuals earlier.[3,4] Propofol-induced priapism in a healthy male is reported herein, the first one in the Indian literature.

## Case Report

A 25-year-old healthy male was noted to have an engorgement of the penis in the emergency room after 75 min of an uneventful reduction of dislocation of the right shoulder with 100 mg of propofol. He denied previous events of priapism, genitourinary disease, substance abuse, genital trauma and erectile dysfunction. He did not experience any sexual fantasies while he was on propofol. There were no features suggestive of hypercoagulable state or malignancy. He was not on any other medications, particularly antipsychiatric or antihypertensive agents or medicinal agents of alternative systems of medicine. Examination revealed an enlarged, edematous, erect penis with tense and tender corpus cavernosa with soft corpus spongiosum and glans. The scrotal contents and the prostate were normal. Systemic examination was unremarkable. Complete blood count, blood chemistry including liver function test and coagulation parameters were within normal limits.

As there was no resolution of priapism after 30 min of using the insulin syringe after successful aspiration of venous blood, 1 mL of 2% lidocaine with epinephrine 1:100,000 was injected into each corpus. Complete detumescence occurred within 10 min. He was discharged and advised to have follow-up with the urologist and orthopedician. He was very reluctant to follow any type of post-reduction immobilization and presented again after 3 days with right shoulder dislocation. He received total doses of fentanyl 100 *μ*g and propofol 50 mg for close reduction. He developed priapism within 15 min, which resolved without any intervention. There was no erectile dysfunction during follow-up of 9 months.

## Discussion

Priapism is a persistent erection of the penis that continues hours beyond or in the absence of sexual desire or stimulation.[5] It is classified into non-ischemic (high flow) and ischemic (low flow) based on the blood flow. Physicians are more likely to consider hematological disorders, malignancy or trauma as a differential diagnosis for priapism. Also, it is essential to consider other medications, including antipsychiatric and antihypertensive drugs and substance abuse that are associated with priapism. Drug-induced priapism belongs to the ischemic (low flow) type, which needs immediate intervention in order to prevent the permanent loss of erectile function.[5] There is paucity of reports on anesthetic agents causing priapism. Previously, there were two independent reports on priapism following propofol administration.[3,4]

Our patient experienced a sustained erection after 75 min of propofol administration and required intervention even after discontinuation of the propofol. He subsequently developed priapism with a lower dose of propofol during rechallenge, which resolved without any therapy. This reaction is dose related and can be labeled as a type A class of adverse effect.[6] It can also be considered as “probable/likely” reaction to propofol as per Naranjo’s causality assessment scale, with a score of 8.[7] Hence, it is justifiable to speculate that the development of priapism in this patient is related to administration of propofol due to the temporal relationship. Moreover, it could not be explained by any other concurrent diseases, drugs, chemical intake or other factors.

Vesta[3] and coworkers confirmed a strong association of propofol to priapism with rechallenge. They also established the dose-response relationship. Fuentes *et al*.[4] reported a case of post-operative priapism in a 7-year-old boy without any hematologic disorder after the administration of propofol, which warranted surgical treatment. Our patient represents the second reported case of propofol-induced priapism in the literature, which was confirmed by rechallenge.[8]

Priapism, following the infusion of 20% concentration fat emulsion as a part of total parenteral nutrition, has been described earlier.[9] Although the exact mechanism is not known, the authors attributed it to fat emulsion-related increased thrombin, erythrocyte aggregation and/or fat embolism. In the present case, the lipid component of propofol is unlikely as the concentration of lipids was low and the total amount of lipids delivered was far negligible. In addition, the present case did not develop systemic hypotension or fall in blood pressure following the propofol administration to account for dose-dependent vasodilation, a remote possible mechanism. The other possibility is the anesthetic effect in the spinal cord that might have blocked the sympathetic vasoconstrictor action or that might have enhanced the parasympathetic vasodilatory action causing an abnormal erection.[10] Propofol-induced alteration of nitric oxide-mediated smooth muscle relaxation[1] might have also had a contributory effect. The modulating effect on GABA_A_ and adrenal steriodogenesis[1,2] effects of propofol might have played an additional role in this state of prolonged erection.[11]

One other possibility is susceptibility of an individual to priapism, either to the difference in the pharmacodynamic and pharmacokinetic effects of propofol formulations[12] or to single neucleotide polymorphisms in the metabolic pathway of propofol.

## Conclusion

The present report highlights the development of low-flow priapism as an adverse effect of propofol, a commonly used anesthetic drug in India, which requires immediate attention in order to alleviate complications and minimize the risk of impotence. Further, extra precautions and monitoring is warranted in patients with associated hematological conditions or other risk factors due to their vulnerability to priapism.

## References

[1] Vasileiou I, Xanthos T, Koudouna E, Perrea D, Klonaris C, Katsargyris A (2009). Propofol: A review of its non-anaesthetic effects. Eur J Pharmacol.

[2] Drug information online. http://wwwdrugscom/mmx/propofolhtml.

[3] Vesta KS, Martina SD, Kozlowski EA (2006). Propofol-Induced Priapism, a Case Confirmed with Rechallenge. Ann Pharmacother.

[4] Fuentes EJ, Garcia S, Garrido M, Lorenzo C, Iglesias JM, Sola JE (2009). Successful Treatment of Propofol-induced Priapism With Distal Glans to Corporal Cavernosal Shunt. Urol.

[5] Yuan J, Desouza R, Westney OL, Wang R (2008). Insights of priapism mechanism and rationale treatment for recurrent priapism. Asian J Androl.

[6] Edwards IR, Aronson JK (2000). Adverse drug reactions: Definitions, diagnosis, and management. Lancet.

[7] Naranjo CA, Busto U, Sellers EM, Sandor P, Ruiz I, Roberts EA (1981). A method for estimating the probability of adverse drug reactions. Clin Pharm Ther.

[8] (1992). WHO. International monitoring of adverse reactions to drugs: Adverse reaction terminology.

[9] Ekstrom B, Olsson AM (1987). Priapism in patients treated with total parenteral nutrition. Br J Urol.

[10] Antognini JF, Saadi J, Wang XW, Carstens E, Piercy M (2001). Propofol action in both spinal cord and brain blunts electroencephalographic responses to noxious stimulation in goats. Sleep.

[11] Andersson KE (2001). Pharmacology of Penile Erection. Pharmacol Rev.

[12] Calvo R, Telletxea S, Leal N, Aguilera L, Suarez E, De La Fuente L (2004). Influence of formulation on propofol pharmacokinetics and pharmacodynamics in anesthetized patients. Acta Anaesthesiol Scand.

